# Toxin Profile of *Gymnodinium catenatum* (Dinophyceae) from the Portuguese Coast, as Determined by Liquid Chromatography Tandem Mass Spectrometry

**DOI:** 10.3390/md13042046

**Published:** 2015-04-13

**Authors:** Pedro R. Costa, Alison Robertson, Michael A. Quilliam

**Affiliations:** 1 IPMA—Portuguese Institute of Ocean and Atmosphere/CCMAR—Centre of Marine Sciences Avenida de Brasília s/n, 1449-006 Lisbon, Portugal; E-Mail: prcosta@ipma.pt; 2 Department of Marine Sciences, University of South Alabama, 5871 University Drive North, Mobile, AL 36688, USA; 3 National Research Council of Canada, Measurement Science and Standards, Biotoxin Metrology, 1411 Oxford Street, Halifax, NS B3H 3Z1, Canada; E-Mail: michael.quilliam@nrc.gc.ca

**Keywords:** paralytic shellfish poisoning, paralytic shellfish toxins, phytoplankton, shellfish, harmful algal blooms, *Gymnodinium catenatum*, liquid chromatography-mass spectrometry

## Abstract

The marine dinoflagellate *Gymnodinium catenatum* has been associated with paralytic shellfish poisoning (PSP) outbreaks in Portuguese waters for many years. PSP syndrome is caused by consumption of seafood contaminated with paralytic shellfish toxins (PSTs), a suite of potent neurotoxins. *Gymnodinium catenatum* was frequently reported along the Portuguese coast throughout the late 1980s and early 1990s, but was absent between 1995 and 2005. Since this time, *G. catenatum* blooms have been recurrent, causing contamination of fishery resources along the Atlantic coast of Portugal. The aim of this study was to evaluate the toxin profile of *G. catenatum* isolated from the Portuguese coast before and after the 10-year hiatus to determine changes and potential impacts for the region. Hydrophilic interaction liquid chromatography tandem mass spectrometry (HILIC-MS/MS) was utilized to determine the presence of any known and emerging PSTs in sample extracts. Several PST derivatives were identified, including the *N-*sulfocarbamoyl analogues (C1–4), gonyautoxin 5 (GTX5), gonyautoxin 6 (GTX6), and decarbamoyl derivatives, decarbamoyl saxitoxin (dcSTX), decarbamoyl neosaxitoxin (dcNeo) and decarbamoyl gonyautoxin 3 (dcGTX3). In addition, three known hydroxy benzoate derivatives, *G. catenatum* toxin 1 (GC1), GC2 and GC3, were confirmed in cultured and wild strains of *G. catenatum*. Moreover, two presumed *N-*hydroxylated analogues of GC2 and GC3, designated GC5 and GC6, are reported. This work contributes to our understanding of the toxigenicity of *G. catenatum* in the coastal waters of Portugal and provides valuable information on emerging PST classes that may be relevant for routine monitoring programs tasked with the prevention and control of marine toxins in fish and shellfish.

## 1. Introduction

The marine dinoflagellate *Gymnodinium catenatum* has received a lot of attention since it was associated with paralytic shellfish poisoning (PSP) in the late 1980s [1,2,3]. This algal species produces paralytic shellfish toxins (PSTs) that can accumulate in a variety of commercially harvested seafood species, such as bivalve mollusks. Consumption of contaminated shellfish may lead to severe poisonings, including fatalities [4,5,6]. The toxicity of PSTs is caused by a high affinity inhibition of voltage-gated sodium channels on the extracellular membrane of nerve cell terminals [7,8]. At low levels of exposure, these toxins can cause numbness of the fingers and extremities, tingling, nausea and vomiting; but at higher doses can result in muscular paralysis and death by respiratory paralysis and cardiovascular shock [9,10].

**Figure 1:**
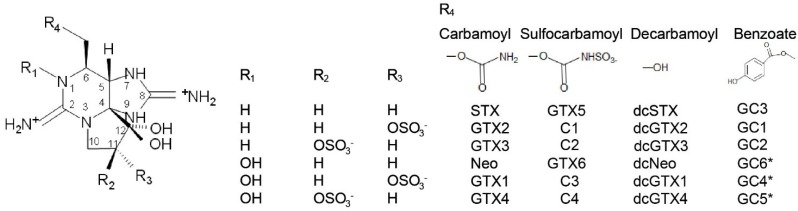
Structure of paralytic shellfish poisoning (PSP) toxins. STX, saxitoxin; GTX, gonyautoxin; Neo, neosaxitoxin; dc, decarbamoyl; GC, *G. catenatum* toxin*.* * Theoretical chemical structures of GC4, GC5, and GC6, suggested by [17].

The most prevalent PSTs can be divided into three groups based on structural differences in their functional groups, namely the carbamoyl, decarbamoyl and sulfocarbamoyl derivatives (Figure 1). Due to these structural differences, each toxin has a slightly different affinity to the binding site of voltage-gated sodium channels, which correlates to differential toxicity [11]. The carbamoyl group, which includes saxitoxin (STX), neosaxitoxin (Neo) and the gonyautoxins (GTX 1–4), are considered the most toxic due to the potent intra-peritoneal toxicity observed in mice [12] and affinity to the sodium channel, as recorded during patch clamp studies [13]. The *N-*sulfocarbamoyl PSTs includes the four C-toxins (C1–4), gonyautoxin-5 (GTX5, formerly B1) and gonyautoxin-6 (GTX-6, formerly B2). Based on intraperitoneal toxicity data, the decarbamoyl group in considered to have moderate toxicity and includes decarbamoyl-STX (dcSTX), decarbamoyl-GTXs (e.g., dcGTX1–4) and decarbamoyl-Neo (dcNeo) [12].

A novel group of PSTs were identified in strains of *G. catenatum* isolated from Australian waters and were named GC1, GC2 and GC3 based on the phytoplankton from which they were isolated [14]. These PSTs have the carbamate side chains substituted with a hydroxybenzoate moiety (see Figure 1). The toxicity of the hydroxybenzoate PSTs (GC toxins) is not well known; however, radio-receptor binding studies using rat brain synaptosomes enriched for sodium channels showed high affinity binding in previous studies [15]. In addition, the phenol ring that is incorporated into the molecule may facilitate transport and bioaccumulation of these compounds due to an increased lipophilicity compared to other PST derivatives [15]. The GC toxins have since been identified in strains of *G. catenatum* isolated from Portugal, Spain, China, Japan and Uruguay [16].

Paralytic shellfish poisoning occurs worldwide, and harmful algal blooms, including those responsible for PSP, appear to be increasing in frequency and intensity [18,19]. PSP outbreaks in Portuguese waters have been associated with blooms of *Gymnodinium catenatum* in the late 1980s to early 1990s, then again after 2005 [2,20,21]. According to the national monitoring program in Portugal, *G. catenatum* were not reported along the Portuguese coast during the 10-year period from 1995 to 2005 [22]. The aims of this study were to fully characterize the toxin profile of *G. catenatum* strains isolated from the NW Portuguese coast before and after the 10-year absence of blooms to determine changes and potential implications for the region. Hydrophilic interaction liquid chromatography tandem mass spectrometry (HILIC-MS/MS) was utilized to determine the presence of any known and emerging PSTs in sample extracts.

## 2. Results and Discussion

### 2.1. Identification of “Classic” PSTs in G. catenatum

**Figure 2:**
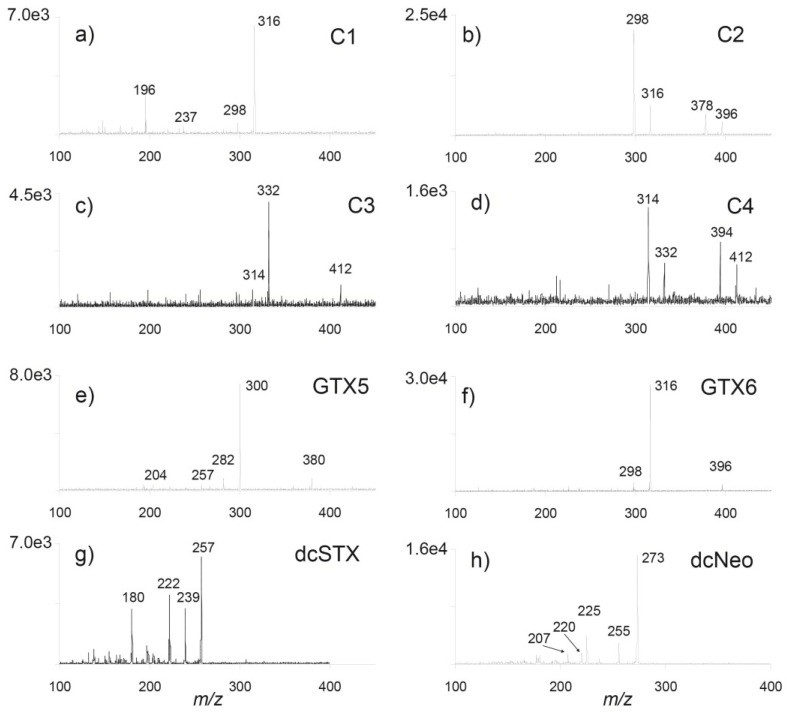
Product ion spectra of paralytic shellfish toxins identified in a *Gymnodinium catenatum* culture isolated from the Portuguese coast in 2007: (**a**) *m/z* 316 of C1; (**b**) *m/z* 396 of C2; (**c**) *m/z* 412 of C3; (**d**) *m/z* 412 of C4; (**e**) *m/z* 380 of GTX5; (**f**) *m/z* 396 of GTX6; (**g**) *m/z* 257 of dcSTX; and (**h**) *m/z* 273 of dcNeo. The [M + H]^+^ ions were selected from full scan analyses and fragmented to achieve the maximum abundance of product ions.

**Figure 3:**
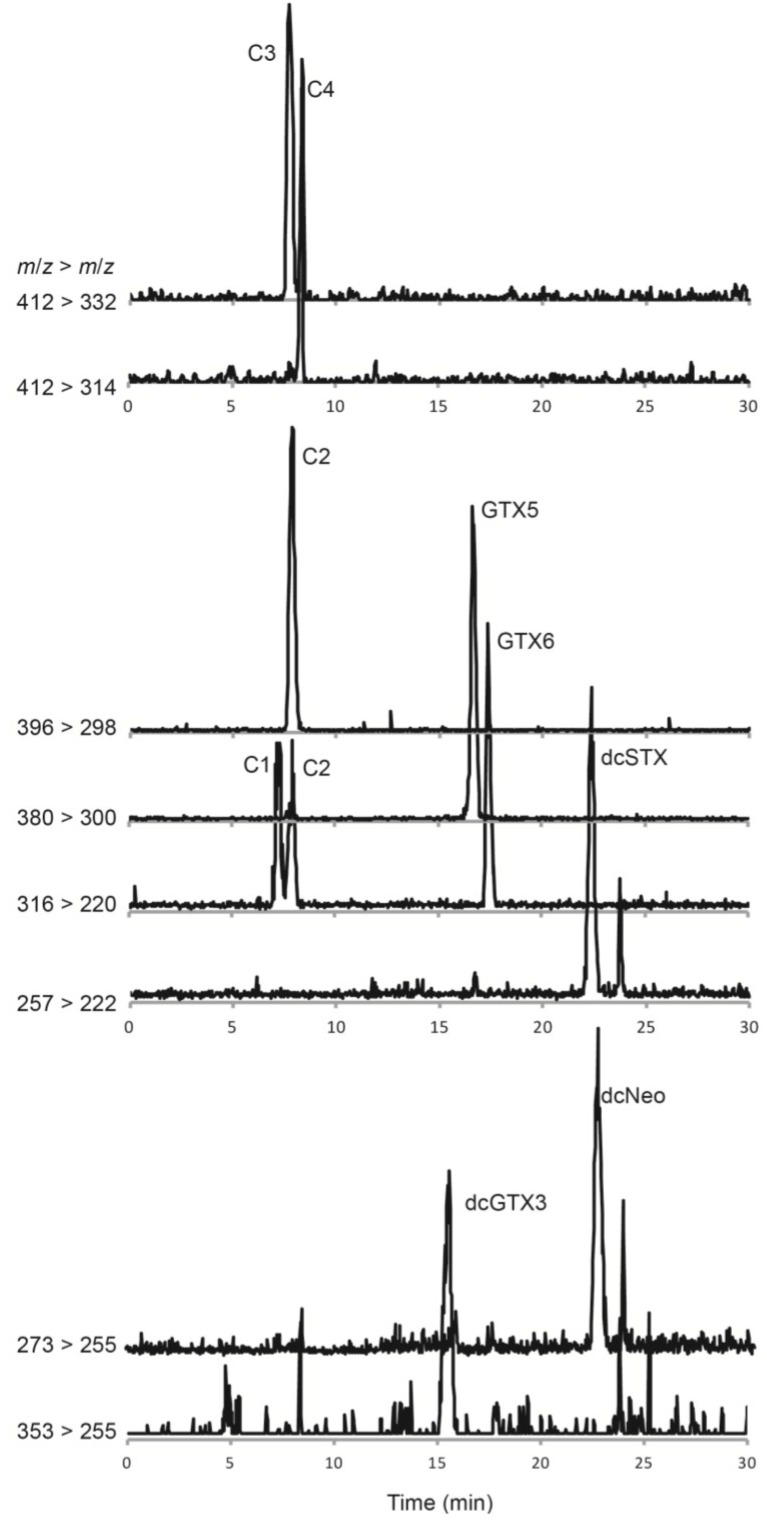
Hydrophilic interaction liquid ion chromatography mass spectrometry analysis of paralytic shellfish toxins in a *Gymnodinium catenatum* culture isolated from the Portuguese coast in 2007. Multiple reaction monitoring in positive polarity was used to identify toxin derivatives. At least two confirmatory ion transitions were monitored for each paralytic shellfish toxin (PST) derivative. The primary transition ions are shown for all confirmed toxins in the samples: *m/z* 257 > 222 for dcSTX, *m/z* 273 > 255 for dcNeo, *m/z* 316 > 220 for C1 and GTX6, *m/z* 353 > 255 for dcGTX3, *m/z* 380 > 300 for GTX5, *m/z* 396 > 298 for C2, *m/z* 412 > 314 for C4 and *m/z* 412 > 332 for C3 (see Table 2 for details on the MS/MS parameters).

The profile of PSTs in a cultured strain of *Gymnodinium catenatum* isolated from Cascais Bay in 2007 and a seawater sample collected during a bloom of *G. catenatum* off Aveiro also in 2007 was characterized via HILIC-MS/MS. After having optimized the MS parameters, different MS modes were used for the analysis of the algae extracts. Starting from the full scan mode, one can obtain information about the ions formed in the ion source, which is necessary for further developing MRM acquisitions. The *G. catenatum* isolated from Portuguese waters was found to produce at least 14 compounds that are structurally related with saxitoxin. Data obtained by tandem mass spectrometry revealed a profile of PSTs in wild and cultured strains constituted by *N-*sulfocarbamoyl and decarbamoyl toxin analogues, namely C1–4, GTX5, GTX6, dcSTX, dcNeo and dcGTX3 (Figure 2). The ion transitions giving optimal results are illustrated in Figure 3. Identification was based on a match of retention times, MRM transition ratios and full scan spectra with those of standards. Carbamate toxins, such as STX or Neo, were not detected in the samples analyzed. 

### 2.2. Identification of Hydroxybenzoate PSTs in Portuguese G. catenatum

**Figure 4:**
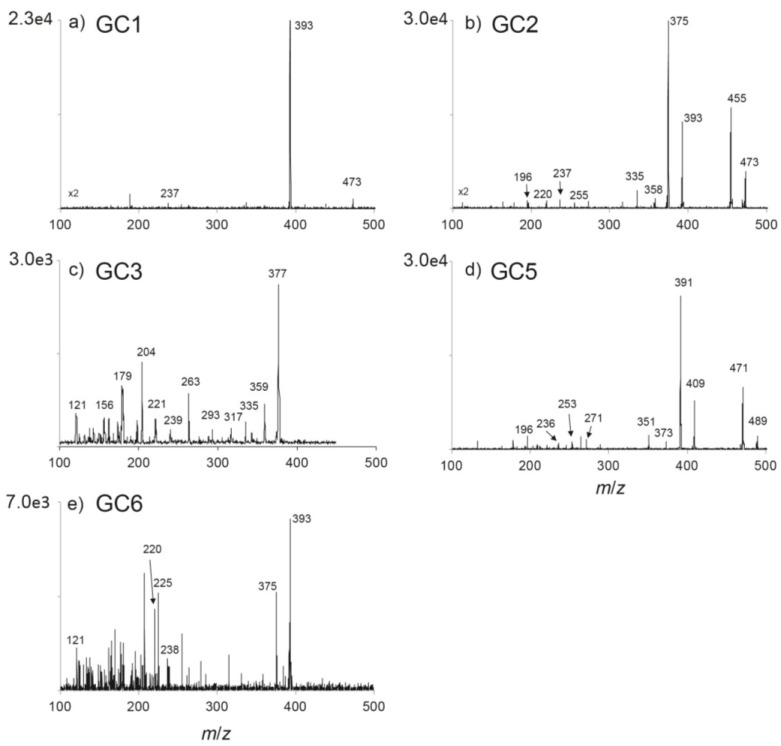
Product ion spectra of GC toxins identified in a *Gymnodinium catenatum* culture isolated from the Portuguese coast in 2007: (**a**) *m/z* 473 of GC1; (**b**) *m/z* 473 of GC2; and (**c**) *m/z* 377 of GC3; (**d**) *m/z* 489 of GC5; and (**e**) *m/z* 393 of GC6. The [M + H]^+^ ions were selected from full scan analyses and fragmented to achieve the maximum abundance of product ions.

**Figure 5:**
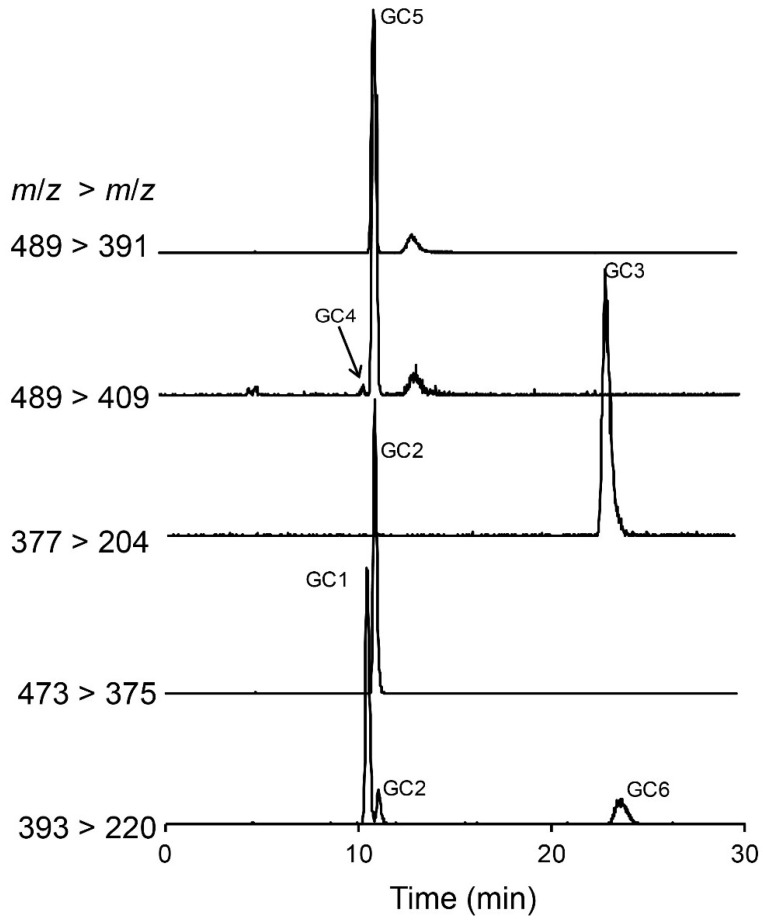
Hydrophilic interaction liquid ion chromatography-mass spectrometry analysis of paralytic shellfish toxins in a *Gymnodinium catenatum* culture isolated from the Portuguese coast in 2007. Multiple reaction monitoring in positive polarity was used to identify hydroxybenzoate PST analogues GC1–6 that were detected and confirmed in a *Gymnodinium catenatum* culture isolated from the Portuguese coast. Primary ion transitions are shown: *m/z* 377 > 204 for GC3; *m/z* 393 > 220 for GC1 and GC6; *m/z* 473 > 375 for GC2, *m/z* 489 > 409 for GC4 and 5, *m/z* 489 > 391 for GC5 (see Table 2 for details on the MS/MS parameters).

In addition to the above reported *N-*sulfocarbamoyl and decarbamoyl toxins, the three hydroxybenzoate derivatives described by [14], *i.e.*, GC1–3, were also identified in the extracts of *G. catenatum*. The structural information from the MS/MS product ion spectrum revealed three or more diagnostic ion fragments for each compound (Figure 4). The MRM method was then optimized based on product ion scans. The ion transitions giving optimal results are illustrated in Figure 5. Identification of GC1–3 was confirmed by a good match of retention times, MRM transition ratios and full scan spectra with those of standards produced in the earlier study [14].

A late eluting peak in the GC1 ion transition was observed, which, after subsequent full scan and product ion scans, was revealed to be due to GC6, as theoretically reported by [17]. The MS/MS product ion spectrum of the [M + H]^+^ ion of GC6 shows a dominant fragment at *m/z* 375, which is due to the loss of a water molecule (Figure 4). Additional fragments are coincident to GC1 and GC2 isomers allowing detection of GC6 in MRM transitions, such as *m/z* 393 > 220 (Figure 5). In support of the structure being assigned as GC6, the shift of *m/z* 204 in the GC3 spectrum to *m/z* 220 in the new compound supports the location of the extra hydroxyl being in the main ring and not on the side chain. The most likely location that conforms to other PST structures is on N1, *i.e.*, a Neo analogue. In addition, the retention time of the compound is slightly greater than GC3, which is consistent with the GC6 structure, as evidenced by the retention time relationship between Neo and STX. 

During full scan analysis of the algae extract, an [M + H]^+^ ion at *m/z* 489 was also observed. This most likely corresponds to the theorized GC5 derivative [17]. Characteristic fragmentation during product ion scans confirmed that this was highly likely to be GC5 (see Figure 4). The MS/MS product ion spectrum showed prominent fragment ions at *m/z* 471 (loss of H_2_O), *m/z* 409 (loss of SO_3_) and *m/z* 391 (loss SO_3_ + H_2_O). In support of the structure being assigned as GC5, the shift of *m/z* 220 in the GC2 spectrum to *m/z* 236 in the new compound supports the location of the extra hydroxyl being in the main ring and not on the side chain. The most likely location that conforms to other PST structures is on N1, *i.e.*, a GTX4 analogue. The retention time of this compound is slightly greater than GC2, which is also consistent with the GC5 structure.

Dinoflagellates have been shown to produce β-epimer forms of PSTs in higher abundance than α-epimers, which are thought to arise through epimerization [23]. Our data were consistent with this assumption with C2, C4, dcGTX3, GC2 and GC5 identified in the *G. catenatum* extracts (see Figure 3). Due to epimerization within living cells or under extraction conditions, the α-analogues can also be detected [23,24]. In our study, the α-analogues C1 and GC1 of the abundant C2 and GC2 compounds were detected in the algal extract. The level of dcGTX3 in our samples was very low, so as expected, the α-analogue, dcGTX2 was below our detection limits. Similarly, a peak corresponding to the putative GC5 was identified, but only trace levels of GC4 were found. A small peak in the MRM transition *m/z* 489 > 409 was observed to elute just before GC5 that corresponds to the putative GC4 (see Figure 5).

One of the aims of this study was deciphering the presence of benzoate derivatives. Particular heed was taken to the search for isoforms with the hydroxyl group replaced by a hydroxyl sulfate group in the benzoate side chain. These compounds would be designated as GC7–12. However, MS/MS analysis did not show their presence in the extracts analyzed, though the theoretical existence of more than 18 benzoate analogues was previously proposed [17].

### 2.3. Understanding Gymnodinium catenatum Bloom Hiatus in Portuguese Waters

Although it was described for the first time in Gulf of California, Mexico, in 1939 [25], *Gymnodinium catenatum* was only associated with PSP episodes in 1976 in Galicia, NW Spain, and then in 1986 in Portugal [1,2]. This species was since then reported with increasing frequency and geographic distribution, including Australia, Japan, South America (Venezuela, Uruguay) and the tropical Indo-West Pacific (Philippines, Singapore and Malaysia) [26]. Marked differences in the profile of PSP toxins have been observed among these regions [16]. Therefore, PSP toxin phenotypes may be used to understand species introduction, migration or adaptation to environments. 

The European origin of *G. catenatum* remains unclear. Cyst records in dated sediment cores indicate that the arrival of *G. catenatum* to the south Iberian coast, expanding then northwards, was in 1889 ± 10 [27,28]. After the first bloom of *G. catenatum* detected in the Portuguese coast in 1985, the species was recurrently registered in the NW coast until 1992, when blooms of *G. catenatum* were also detected in the south and southwest coast [20,29]. Despite the maintenance of a regular monitoring program for toxic algae, no evident blooms of *G. catenatum* were observed between 1996 and late 2005 [22]. In order to understand the resurgence of *G. catenatum* after a 10-year hiatus of absence, the profile of PSP toxins determined in this study was compared with previous studies where *G. catenatum* strains isolated before 1995 were analyzed (Table 1). In the present study, the dominant compounds, in terms of molar fraction, were GC1–3, C1, C2, and GTX5 in both wild and cultured strains (Table 1). Characterization of the profile of toxins produced by *G. catenatum* isolated from the Portuguese coast was firstly carried out by post-column LC-FLD analysis [30]. At a time when standards and confirmatory methods were limited, the profile of toxins was constituted only by the C1, C2, dcSTX and GTX5. More recently, several *N-*sulfocarbamoyl, decarbamoyl and hydroxybenzoate toxins were determined in a *G. catenatum* strain isolated from the Portuguese coast in 1989 [16]. The toxins identified in these two previous studies are in accordance with the findings of the present study.

**Table 1:** Toxin profile (mol%) from the *Gymnodinium catenatum* culture isolated from Cascais Bay in 2007, a seawater sample collected during a bloom of *G. catenatum* off Aveiro in 2007 and a strain isolated from Aguda in 1989 that was cultured and analyzed by [16].

Toxin	Toxin Cell Quota (fmol/Cell) ^		Toxin Profile (mol%)		
	This Study		This Study		Negri *et al.* (2007)
	Culture	Seawater	Culture	Seawater	Culture (1989)
C1 + 2	6.5	4.3	21.0	22.1	14.5
C3 + 4	0.5	0.4	1.6	2.0	6.0
GTX2 + 3	nd	nd	nd	nd	1.0
GTX5	3.4	6.9	11.0	35.2	12.9
GTX6	1.2	nd	3.8	nd	5.9
dcGTX2 + 3	0.3	nd	0.8	nd	4.0
dcSTX	2.2	nd	7.2	nd	2.5
dcNeo	1.1	nd	3.4	nd	nd
STX	nd	nd	nd	nd	0.4
GC1 + 2	5.9	6.2	19.0	31.6	35.3
GC3	5.0	1.6	16.2	8.3	17.6
GC4 * + 5	1.7	0.2	5.6	0.8	na
GC6	3.2	nd	10.3	nd	na

nd: not detected; na: not analyzed; * trace level GC4 detected, but not in the quantifiable range; ^ LC-MS/MS peak area for individual PSTs was compared to the corresponding toxin standard curves to calculate the concentration (µM), then standardized to the combined total fraction as a percentage to calculate the mol% as detailed in the Experimental Section.

Although variations of the PST profile in terms of the relative abundance of each toxin are expected to occur with *G. catenatum* growth phases, cell culture age or nutrient availability [31,32], there is consistency of the suite of toxins within populations [33,34]. The same suite of PSTs was found in the culture of a strain isolated in 2007 (this study), in a seawater sample collected during a bloom of *G. catenatum* in 2007 (this study) and strains isolated before the 10-year hiatus, as reported by [16] and by the early studies performed by [30]. Using the toxin profile as the chemical signature, the recent resurging of *G. catenatum* in the Portuguese coast should not be viewed as a result of a reintroduction of the species from other regions, such as for example via ship ballast waters. The explanation for this discontinuing occurrence still needs to be elucidated. One hypothesis may be related to an important feature in the life-cycle of *G. catenatum*, which is the formation of a non-motile resting cyst that is resistant to degradation and may persist to be viable in sediments [35,36]. 

### 2.4. Management Implications

From the pool of the toxins detected, only six are routinely monitored in Portuguese shellfish via the conventional HPLC-FLD method developed by Lawrence [37]. Attempting to move away from animal testing, the HPLC-FLD method was accepted by European directives as an alternative to the mouse bioassay (MBA) for the determination of PSP toxins in shellfish [38]. However, determination of the entire range of toxins present in the matrix is required for the assessment of the risk associated with the human consumption of shellfish. In this study, the presence of toxins for which commercial standards were not previously available, *i.e.*, C3, C4, GTX6 and the GC toxins, GC1, GC2, GC3, GC4, GC5 and GC6, were confidently identified in *G. catenatum* extracts. These toxins are not part of the routine PSP monitoring in many regions due to the lack of analytical standards, but they may be important contributors to the composite toxicity of shellfish. Fortunately, we were able to use certified reference materials for 12 of the 18 PST derivatives investigated during this study. Although the *N-*sulfocarbamoyl analogues C3 and C4 and GTX6 are considered less toxic than most other PSTs [12,39], the high concentrations reached in mussels exposed to *G. catenatum* blooms showed a significant contribution of these low potency analogues to the PSP toxicity of the sample in our previous study [40]. Very little data exist on the bioaccumulation of GC toxin analogues, and no data exist for GC4–6 [6,41]. The observation of high levels of GC toxins found in *G. catenatum* from the Portuguese coast in this study requires an extensive follow-up investigation of trophic transfer in the marine food web, so that we may assess the potential human health risk. However, to date, only one study investigated the presence of GC toxins in shellfish and in sardine viscera [42]. In this study, shellfish samples collected from the Portuguese coast revealed that up to 18% of the total PST molar fraction was due to GC toxins, but quantification and confirmation was not possible, due to the lack of reference standards [42]. The less polar hydroxybenzoate moiety of GC toxins would favor its bioaccumulation and reduce their elimination rate [14,15]. Further research is needed to understand the dynamics of GC toxins accumulation, elimination and, indeed, their contribution to composite mammalian toxicity.

It is also important to review the detection methods used for routine analysis of food control. The reverse-phase SPE clean-ups used to remove interferences on the HPLC methods will result in the loss of these hydrophobic toxins, resulting in a potential underestimation of composite toxicity. More recently, another method that is an alternative to the MBA, the receptor binding assay for PST [43,44], has become an official method of the International Association of Analytical Chemists (AOAC International) (AOAC 2011.27). This biochemical method may be a good candidate for complex matrices containing multiple toxins, since its requirements in terms of standards are very simple, needing only for routine analysis the STX radiolabeled calibrant. A modified version of this method was used to assess the affinity of GC toxins 1–3 to the rat brain sodium channel [15]. These investigators found that the GC toxins had a high affinity (nanomolar range) in the receptor binding assay and concluded that an expected increase in lipophilicity gained by the addition of the phenol ring in the GC saxitoxin structure may increase the potential for bioaccumulation in seafood species. Discrimination of the toxin profile was not achieved through this method; however, there appears a good correlation between biological activity and toxicity for the entire range of PST [43].

## 3. Experimental Section

### 3.1. Reagents and Standards

All organic solvents were HPLC or LCMS grade. Water was distilled and passed through a MilliQ water purification system. Formic acid (LCMS grade) and acetic acid (AR grade) were purchased from Fisher Scientific (Pittsburgh, PA, USA). The following certified reference materials for PSTs were provided by the National Research Council (NRC) of Canada (Halifax, NS, Canada) and used throughout this study: CRM-STX-e, CRM-Neo-b, CRM-GTX2&3-b, CRM-GTX1&4-b, CRM-dcSTX, CRM-dcGTX2&3, CRM-GTX5-b, CRM-C1&2 and CRM-dcNeo-b. Reference standards for three GC-toxins (GC1–3) were also prepared by the NRC group and used throughout this study for the generation of standard curves and optimization of MS methods. The molar concentrations of GTX6, C3, C4, GC4, GC5 and GC6 was estimated using standards of structurally similar compounds, namely GTX5, C1, C2 and GC3, respectively. A similar molar and peak area response was assumed for these compounds. 

### 3.2. Sample Collection and Culture

Surface seawater samples were collected during *G. catenatum* blooms along the NW Portuguese coast in 2007 [45]. Seawater samples containing the highest cell density of 2.5 × 10^4^ cells of *G. catenatum* L^−1^ were chosen for this study. A 1.5 L volume of seawater was vacuum filtered on 0.45-µm pore size mixed cellulose ester (HA) membrane filters (Durapore, EMD Millipore Inc., Boston, MA, USA) and frozen at −20 °C, as previously described [46]. Cultured strains were isolated from a *G. catenatum* bloom in Cascais Bay, Portugal, in September, 2007. Cells were grown in f/2 medium at 18 °C and 250 µE m^−2^ s^−**1**^ light with a 12:12 light:dark cycle. Cells were harvested from the 2-L culture by centrifugation at 2000× *g* for 5 min at 15 °C. The cell density of the cultured strain was 6.6 × 10^5^ cells L^−1^.

### 3.3. Chemical Extraction of PSTs

Cell pellets and filters were extracted in 5 mL of 0.05 M acetic acid using a probe sonicator at 25 W, 50% pulse duty cycle (Branson Sonifier 450, Danbury, NH, USA) for 4 min on ice. Cell lysis was confirmed using light microscopy. The extracts were then centrifuged at 4000× *g* for 10 min, and the supernatant cleaned by solid phase extraction (SPE) using an octadecyl bonded phase silica (Supelclean LC-18 SPE cartridge, 3 mL, Supelco, Sigma Aldrich, St. Louis, MO, USA) cartridge, which had been pre-conditioned with 6.0 mL methanol followed by 6.0 mL water. A 1.0-mL aliquot of the extract was loaded onto the column and PSTs eluted with 2.0 mL of water as reported by [37]. Following a wash with 2.0 mL of water, the cartridges were then subjected to a second elution with 80% v/v aqueous methanol to capture any hydrophobic GC toxin derivatives. This fraction was then evaporated to dryness under N_2(g)_ at room temperature and re-constituted in 0.5 mL water.

### 3.4. Liquid Chromatography Tandem Mass Spectrometry

Toxin analysis was performed on a Waters Acquity UPLC system coupled to a Waters Quattro Micro triple quadrupole mass spectrometer. The analytical method employed during this study was modified from similar protocols reported by [47,48]. A 5-µm amide bonded silica (TSK-gel Amide-80^®^) column (250 mm length × 2.0 mm i.d.) with a guard column (2 mm × 1 cm) with the same stationary phase from Tosoh Bioscience (Grove, CA, USA) was selected. The column was maintained at 30 °C, and a sample injection volume of 10 µL was used. Toxins were eluted at a flow rate of 0.2 mL/min with a normal phase gradient from 65% B to 35% B in 30 min, followed by a 5-min hold at 35% B, where Mobile Phase A consisted of 50 mM formic acid in water (pH = 2.6) and Mobile Phase B consisted of 95% aqueous acetonitrile containing 50 mM formic acid. Analyses were performed in positive ion mode using full scan, product ion scan and multiple reaction monitoring (MRM) experiments. The following MS source conditions were used for analysis: capillary voltage at 2.0 kV, source temperature 125 °C, desolvation temperature 390 °C, cone gas flow 200 L h^−1^, desolvation gas flow at 500 L h^−1^, capillary voltage 2 kV. Transition ion selection for MRM experiments was determined following product ion scans, with cone voltage and collision energies optimized for each transition ion pair using NRC-certified reference materials and reference standards (Table 2). These standards were used in the method development to optimize the mass spectrometer conditions and spectra for survey scans (MS and MS/MS) and MRM analyses. In addition, reference standards were used to generate external calibration curves, for direct comparison of retention times and comparison of transition ion ratios in this study. The limit of detection (LOD, *S/N* = 3) was calculated at 0.02 nM for STX. The concentration of GTX6, C3, C4, GC4, GC5 and GC6 were estimated assuming an equal molar and peak response of structurally similar analogs, since certified reference standards were available for just 12 of the 18 toxins surveyed in this study. In these instances, the concentration was determined based on the calibration curves of the most structurally similar analog (*i.e.*, adjusted to the known molecular weight of the toxin to determine the concentration. Minor sample matrix effects (e.g., ionization suppression and retention time shifts) were resolved with sample dilution (1:5 ratio) in the mobile phase, with resultant within- and between-day residuals for a retention time >4%. The concentration of individual PST derivatives is expressed on a per cell basis and as a molar percentage of the combined PST mixture identified in phytoplankton cultures and seawater. Data were acquired and post-processed using Waters MassLynx™ software, v. 4.1.

**Table 2:** Primary and secondary transition ion pairs for paralytic shellfish toxins (PSTs) analyzed by multiple reaction monitoring in this study, except those that were in low abundance or not detected in algae extracts. Optimized collision energy and declustering potential for each transition ion pair is provided.

Toxin	Transition Ion Pair (*m/z*)	Collision Energy (eV)	Declustering Potential (V)
dcSTX	257 > 222	19	35
	257 > 239	10	30
dcNeo	273 > 255	20	35
	273 > 225	25	35
STX	300 > 204	20	35
GTX3	316 > 220	20	35
dcGTX3	353 > 255	20	35
GTX5	380 > 300	14	20
	300 > 204	20	35
C2	396 > 298	10	40
	396 > 316	15	45
C1	316 > 220	20	35
	396 > 316	15	45
GTX6	316 > 220	20	35
	396 > 316	15	45
C4	412 > 314	10	40
	412 > 332	15	45
C3	412 > 332	15	45
GC1	393 > 320	15	20
	473 > 393	15	20
GC2	473 > 375	15	20
	473 > 455	15	20
GC3	377 > 204	25	40
	377 > 359	20	30
GC4	489 > 409	20	30
GC5	489 > 391	20	30
	489 > 471	15	20
GC6	393 > 220	20	40
	393 > 375	15	20

## 4. Conclusions

The HILIC-MSMS analyses performed to determine the profile of PSP toxins produced by *G. catenatum* from the Portuguese coast confirmed the dominant presence of the *N-*sulfocarbamoyl and hydroxybenzoate PSP analogues. The characterized profile resembles that of *G. catenatum* isolated in the Portuguese coast before the 10-year period of *G. catenatum* absence, suggesting that resurgence of this cryptogenic dinoflagellate is not a result of reintroduction or new import from other regions. Two new hydroxybenzoate analogues, GC5 and GC6, were characterized for the first time through tandem mass spectrometry. Based on the estimated molar fraction, these benzoate analogues were the major constituents of the *G. catenatum* strains analyzed, and they pose a new challenge from a monitoring and regulatory perspective. The reduced knowledge of their toxicity, the lack of analytical certified standards for GC toxins in addition to the diverse nature, *i.e.*, hydrophilic and hydrophobic, of the entire range of compounds found in *G. catenatum* makes it necessary to further investigate the fate of these compounds in the food web and to develop appropriate screening and detection methods.
